# Monitoring health related quality of life in survivorship care of young adult survivors of childhood cancer using web-based patient-reported outcome measures: *survivors’ and health care practitioners’ perspectives on the KLIK method*

**DOI:** 10.1007/s11136-023-03504-z

**Published:** 2023-08-24

**Authors:** Anne Maas, Heleen Maurice-Stam, Marloes H. van den Heuvel, Maria M. W. Koopman, Jaap G. den Hartogh, Leontien C. M. Kremer, Martha Grootenhuis

**Affiliations:** 1 Princess Máxima Center for Pediatric Oncology, Heidelberglaan 25, 3584 CS Utrecht, The Netherlands; 2 Vereniging Kinderkanker Nederland, De Bilt, The Netherlands; 3 Department of Pediatrics, Amsterdam UMC, University of Amsterdam, Amsterdam, The Netherlands

**Keywords:** Childhood cancer survivors, Patient reported outcome measures, Health-related quality of life, Survivorship care, Digital healthcare, Monitoring

## Abstract

**Purpose:**

The KLIK method is a tool to systematically monitor and discuss Health Related Quality of Life (HRQOL) in clinical practice. It has been successfully used in clinical practice in The Netherlands, and has recently been implemented in survivorship care for young adult childhood cancer survivors (CCSs). This study evaluates implementation fidelity and satisfaction of CCSs and healthcare practitioners (HCPs) with the KLIK method in survivorship care.

**Methods:**

CCSs’ HRQOL was monitored using the KLIK questionnaire (PedsQL generic 18–30 years). In a mixed-methods design, implementation fidelity was based on registrations, and user satisfaction was assessed with evaluation surveys (CCSs) and semi-structured interviews (CCSs, HCPs). Descriptive statistics and qualitative analysis methods were used.

**Results:**

A total of 245 CCSs were eligible for the study. Fidelity was 79.2% (194/245) for registration in the KLIK PROM portal, 89.7% (174/194) for completed KLIK questionnaires, 74.7% (130/174) for its discussion during consultation. Of the eligible CCSs, 17.6% (43/245) completed the study evaluation survey. Five CCSs and HCPs were invited for an interview and participated. CCSs (7.7/10) and HCPs (7.5/10) were satisfied with the KLIK method. Reported facilitators included increased insight into CCSs’ functioning, improved preparation before, and communication during consultation, without lengthening consultation duration. Barriers included CCSs not always completing KLIK questionnaires, incomplete content of the KLIK questionnaire, and the need for customization for CCSs with cognitive disabilities.

**Conclusion:**

The KLIK method is a feasible and valuable tool to systematically monitor and discuss HRQOL in survivorship care. Integration of the KLIK method within the organization is essential, with structural support in reminding CCSs to complete questionnaires.

**Supplementary Information:**

The online version contains supplementary material available at 10.1007/s11136-023-03504-z.

## Introduction

Today we face a growing population of childhood cancer survivors (CCSs) thanks to survival rates exceeding 80% [1–3]. Most CCSs are advised to attend life-long survivorship care to screen for late consequences of childhood cancer and its treatment [4–7]. However, the cancer experience may also affect CCSs’ psychological well-being [8]. This warrants monitoring psychosocial issues and Health Related Quality Of Life (HRQOL) in survivorship care to identify and discuss problems with health care practitioners (HCPs), and to offer psychosocial support [9, 10].

The use of PROMs (Patient Reported Outcome Measures) in clinical practice to systematically monitor HRQOL with standardized, validated questionnaires has gained relevance [11]. PROMs have been used in a variety of settings, including both children and adult cancer (survivorship) care [12]. PROMs facilitate the recognition of (mental) health problems, and improve HRQOL, treatment outcomes, physician communication, satisfaction with care, and self-management in patients [12–19].

**Fig. 1:**
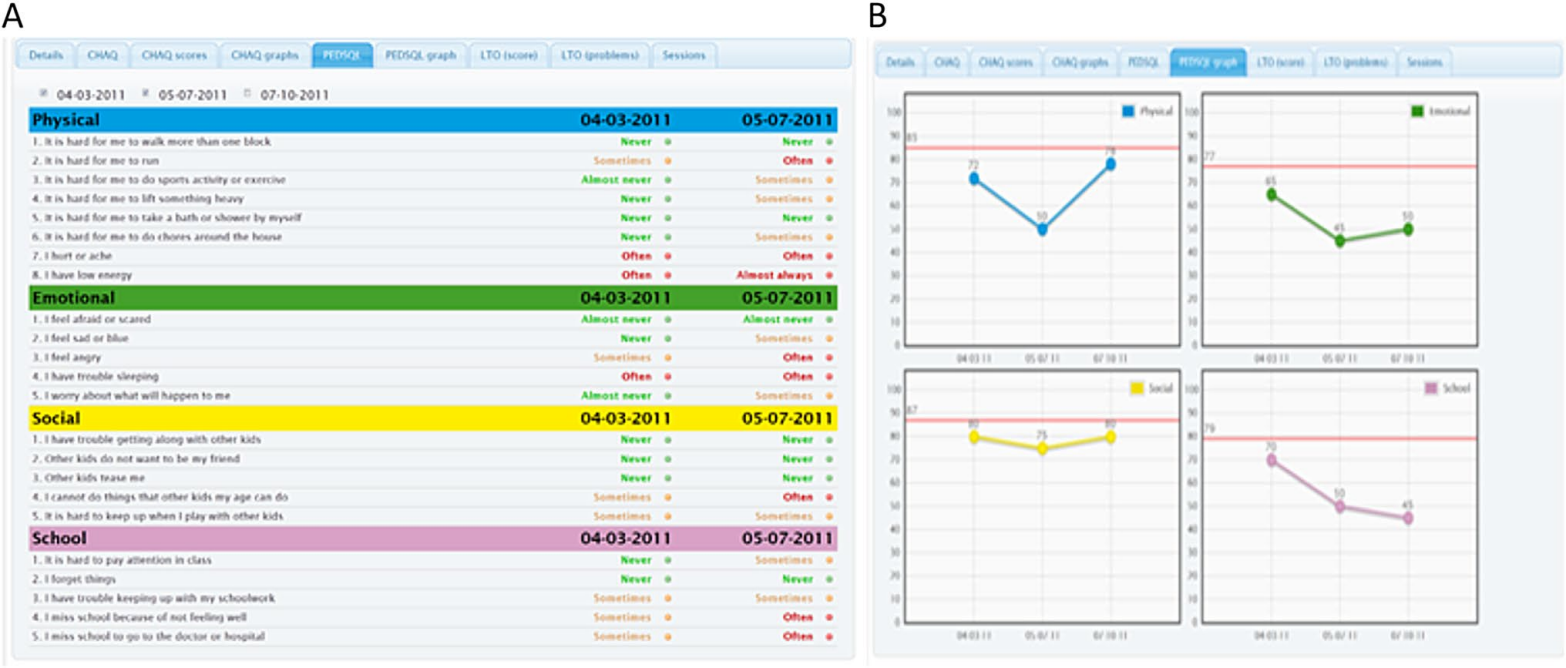
**A** KLIK ePROfile: example of literal feedback on the individual items of the PedsQL for a patient in active treatment, and **B** KLIK ePROfile: example of graphical feedback of the PedsQL over time for a patient in active treatment, including norm lines

To enable the use of PROMs in clinical practice, the KLIK method was developed, for which the KLIK PROM portal (www.hetklikt.nu, www.klik-uk.org) is being used. The KLIK PROM portal is an online portal in which HRQOL is monitored using PROMs [20, 21]. Patients complete standardized, validated questionnaires in the online portal to create a two-part schematic digital view (items and sum-scores), called the ePROfile (Fig. 1). The ePROfile is fed back to the HCP via a dashboard in the medical file of the patient. The ePROfile can be used to systematically monitor and discuss HRQOL during the consultation [22]. Previous studies have shown that the KLIK method facilitates HCPs’ assessment and discussion of psychosocial problems while not significantly lengthening clinic visit time [20]. Additionally, it enhances HCPs’ satisfaction with the consultation [18]. Both patients, parents and clinicians appeared satisfied with the KLIK method and reported benefits such as increased insight into patient’s functioning, and improved communication and detection of HRQOL problems. Barriers were also reported such as HCPs not discussing PROMs and patients not completing them [23–25].

The KLIK method has a long history from development [20, 21], effectiveness studies [18, 26] to implementation [23]. Since 2011 the KLIK method has been implemented in over 20 hospitals in The Netherlands [22] including the Princess Máxima Center for Pediatric Oncology (Máxima) where the KLIK method is used successfully in childhood cancer patients and CCSs aged < 18 [23]. The next step was the implementation in survivorship care in Máxima’s LATER outpatient clinic for young adult CCSs (aged 18–30). Previous studies on perspectives on the KLIK method focused on underage patients and their parents in children’s hospitals, and their clinicians [24, 25]. In the present study we address the perspectives of young adult CCSs and HCPs in survivorship care. As the frequency of visits in survivorship care is lower than in patients receiving active treatment, it is important to study whether previous results can be generalized to this group. The mixed methods design of this study, combining actual user data with questionnaires and interviews provides a complete and in-depth picture of the use of the KLIK method in survivorship care. This study aims to evaluate HRQOL monitoring using the KLIK method in survivorship care of young adult CCSs by assessing implementation fidelity, and user satisfaction from the perspectives of CCSs and HCPs. The results of this study give insight into the possibilities of using PROMs in survivorship care including possible barriers and facilitators.

## Methods

### The KLIK method

The aim of the KLIK PROM portal is to systematically monitor and discuss HRQOL in clinical practice. As an integral part of the KLIK method, the KLIK questionnaire is used as a PROM in clinical practice. The KLIK questionnaire consists of two parts. Firstly, the Pediatric Quality of Life Inventory for Young Adults (PedsQL YA): a self-reported measure of HRQOL for young adults (age 18–30). It consists of 23 items comprising four functioning scales: Physical, Emotional, Social, and School/work [27, 28]. Secondly, an open field question is included allowing CCSs to note any questions or issues they may have for discussion with the HCP. The KLIK questionnaire is offered to young adult CCSs invited to the LATER outpatient clinic as part of regular care.

CCSs were invited to use the KLIK PROM portal via their hospital appointment letter. As part of the KLIK method, the use of the portal was facilitated by a specialized KLIK team in the Máxima. If CCSs did not register in the KLIK PROM portal a week prior to their consultation, they were called by the KLIK team to provide them with support in this process. Also, the KLIK team answers questions that CCSs or HCPs may have.

Several aspects were taken into account in the implementation of the KLIK method in the LATER outpatient clinic for young adult CCSs. As literature points to the importance of key stakeholders in the implementation process [29, 30], a multidisciplinary implementation team was formed consisting of researchers, HCPs from the LATER outpatient clinic, KLIK team members, and one CCS to guarantee patient participation. The team assisted in streamlining procedures regarding, e.g., the KLIK invitation letter, the involvement of all professions working with the KLIK method, and the selection of the KLIK questionnaire. HCPs received training in the form of a 1.5 h workshop on essential aspects of working with the KLIK method, such as how to access and use the portal, and how to correctly interpret the KLIK ePROfile. Finally, all relevant professions (planners, doctor’s assistants, HCPs) were informed about the KLIK method, its importance, and their tasks.

### Study design and procedures

The present study on the KLIK method is an observational study with a mixed methods design. Implementation fidelity, defined as the percentage of invited CCSs that registered in the KLIK PROM portal, the percentage of registered CCSs that completed the KLIK questionnaire, and the percentage of completed KLIK questionnaires discussed during the consultation, was assessed quantitatively. During the study period (September 30, 2021–January 28, 2022), data from the KLIK PROM portal were used to measure the amount of eligible CCSs that registered in the portal, and completed the KLIK questionnaire. CCSs aged 18–30, who had a consultation at Máxima’s LATER outpatient clinic during the study period were eligible unless they were cognitively unable to complete the KLIK questionnaire or had canceled their consultation and therefore could not have gained experience with the KLIK method. We also excluded CCSs if they had previously used the KLIK method as their preexisting opinions about the KLIK method based on their experience in the context of active treatment may influence their opinion about the KLIK method in the context of survivorship care. Finally, we excluded CCSs if the KLIK team, due to high workload, had been unable to remind them to complete the KLIK questionnaire. These criteria were set to ensure an evaluation focused on regular care including assistance of the KLIK team in the specific context of survivorship care. Notes of the HCPs in CCSs’ files were examined to determine if the questionnaire was discussed during the consultation.

CCSs’ user satisfaction was assessed with an evaluation survey. After their consultation, eligible CCSs were invited by mail to participate in the study.

Participating CCSs registered in the KLIK PROM portal completed the evaluation survey in the portal. CCSs not registered received a pencil/paper survey. CCSs could leave their e-mail address and phone number in the survey if they were willing to take part in an additional interview. Semi-structured telephone interviews (10–15 min) with in-depth questions about the answers given in the evaluation survey were held with a selection of CCSs until data saturation was reached meaning that no new barriers and facilitators were mentioned. The selection of CCSs was based on satisfaction (low/high) and use of the KLIK PROM portal (yes/no).

HCPs’ user satisfaction was assessed with semi-structured interviews. HCPs who had worked with the KLIK method at Máxima’s LATER outpatient clinic during the study period were invited by mail for a (face-to-face) interview (20–30 min). HCPs with insufficient experience, i.e., they had discussed the KLIK questionnaire with < 10 CCSs aged 18–30, were excluded.

Informed consent was obtained from all study participants. Approval for the study was granted by the Clinical Research Committee of the Máxima (June 14, 2001, no 2021-007). We used the SRQR (Standards for Reporting Qualitative Research) checklist to guide the reporting of the qualitative part of the study [31].

### Measures

The evaluation survey for CCSs to assess their satisfaction with the KLIK method was based on a previously used survey to evaluate the KLIK method in patients and parents [24]. One HCP and one CCS from the KLIK implementation team evaluated the survey to ensure that it was suitable for CCSs. CCSs who had used the KLIK method received a survey about their overall satisfaction with the KLIK method, and their satisfaction with different aspects of the KLIK method, namely the usability of the KLIK PROM portal, the content of the KLIK questionnaire, the discussion of the KLIK ePROfile, and the influence of the KLIK method on the consultation (Fig. 3). Overall satisfaction of CCSs was measured on a 0 (not satisfied) to 10 (very satisfied) scale. Satisfaction with several aspects of the KLIK method was assessed on a 5-point Likert scale and with open questions. CCSs who had not used the KLIK method received a separate version of the survey, asking for reasons for not registering in the KLIK PROM portal or not completing the KLIK questionnaire.

The topic list for the HCPs interview was based on a previously used survey to evaluate the KLIK method in clinicians [25]. HCPs were asked about their satisfaction on different aspects of the KLIK method (Appendix A).

### Analyses

The data were stored anonymously in a secured database. The quantitative data were analyzed in IBM SPSS Statistics, version 25, using descriptive statistics. The open questions in the survey and the transcript verbatim of CCSs’ interviews were read carefully to detect any aspects not found with the survey. The interviews with HCPs were transcribed verbatim. Relevant parts were highlighted, and then clustered into main and subthemes. The interviews topics were used as guidance in defining themes. The interviews and analysis were conducted by the first author and checked by the second author. Both authors had experience in qualitative data collection and analysis, and they were not involved in the development of the KLIK method nor did they have personal experience in using the KLIK method.

## Results

### Participants

**Fig. 2:**
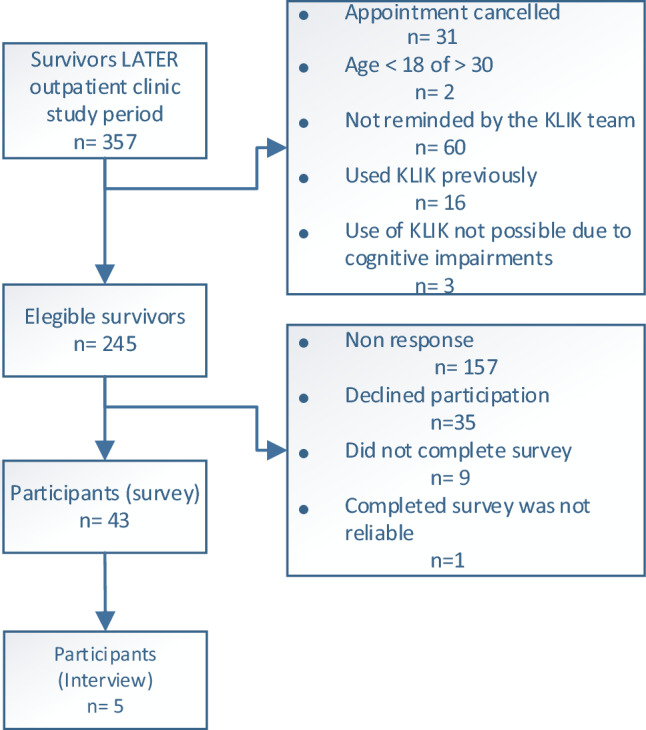
Participants (childhood cancer survivors) flowchart

During the study period, 357 CCSs visited the Máxima LATER outpatient clinic of which 245 CCSs were eligible. Of the 245 eligible CCSs, 43 (17.6%) completed the evaluation survey (Fig. 2). Of the 43 CCSs, 38 CCSs had registered in the KLIK PROM portal and had completed the KLIK questionnaire (Table 1). From this pool of 43 CCSs, five were invited for and participated in an additional interview. Their evaluations are integrated with the evaluation survey outcomes.

**Table 1:** Participants—childhood cancer survivors

	Questionnaires			Interview		
	*N* = 43			*N* = 5		
	*M*	SD	Range	*M*	SD	Range
Age (years)	24.35	2.98	18.35–29.74	25.08	1.68	23.85–27.48
Time since diagnosis (years)	15.67	6.12	4.49–26.61	15.84	7.74	6.29–24.63
	% (*N*)		% (*N*)			
Sex						
Male	46.5 (20)		80 (4)			
Female	53.5 (23)		20 (1)			
Education^a^						
Low	7.3 (3)		0 (0)			
Middle	39.0 (16)		20 (1)			
High	53.7 (22)		80 (4)			
Cancer type						
Hematological	65.1 (28)		80 (4)			
Solid	25.6 (11)		20 (1)			
Central nervous system	9.3 (4)		0 (0)			
Use of KLIK PROM portal						
Not registered	9.3 (4)		20 (1)			
Registered/KLIK questionnaire not completed	2.3 (1)		0 (0)			
Registered/KLIK questionnaire completed	88.4 (38)		80 (4)			
KLIK discussed with HCP	76.3 (29)		100 (4)			
KLIK not discussed with HCP	23.7 (9)		0 (0)			

^a^Education low: primary education, lower vocational education, lower and middle secondary education; middle: middle vocational education, higher secondary education, pre-university education; high: higher vocational education, university

During the study period, six HCPs worked in the LATER outpatient clinic with young adult CCSs. One HCP was ineligible as she had insufficient experience in working with the KLIK method in this age group. All five eligible HCPs participated. Table 2 shows the characteristics of participating HCPs.

**Table 2:** Participants—health care practitioners (*N* = 5)

	*M*	SD	Range
Age (years)	51.00	9.49	39.00–61.00
Experience in LATER outpatient clinic (years)	6.90	10.13	1.50–25.00
	% (*N*)		
Sex			
Female	100 (5)		
Profession			
Nurse specialist	20 (1)		
Internal specialist	20 (1)		
Internal specialist-endocrinologist	20 (1)		
Internal specialist/head of the LATER outpatient clinic	20 (1)		
Pediatric neurologist	20 (1)		
Prior use of KLIK PROM portal			
Yes	20 (1)		
No	80 (4)		

### Implementation fidelity

Of the 245 eligible CCSs, 79.2% (194/245) registered in the KLIK PROM portal, of whom 89.7% (174/194) completed the KLIK questionnaire. In 74.7% (130/174) the completed KLIK questionnaire was discussed by the HCP during the consultation. CCSs who had not used the KLIK method (*N* = 5) reported the following reasons for not doing so: forgotten, not received the KLIK invitation letter, lack of time, or not clear how the KLIK PROM portal worked. One of these CCSs was interviewed and mentioned that the necessity to create an account was a barrier to use the KLIK method, while a more thorough explanation of its purpose could be a motivating factor.

### User satisfaction CCSs

#### Overall satisfaction with the KLIK method

CCSs (*N* = 38) who completed the KLIK questionnaires reported on average an overall satisfaction with the KLIK method of 7.7 (range 2–10). Most CCSs rated the KLIK method ≥ 8 (68.5%), but a few (3 CCSs) gave a score < 6 because of too much e-mail reminders to complete the KLIK questionnaire or because their answers to the questionnaire were not visible to or not discussed by the HCP.

#### Usability of the KLIK PROM portal

**Fig. 3:**
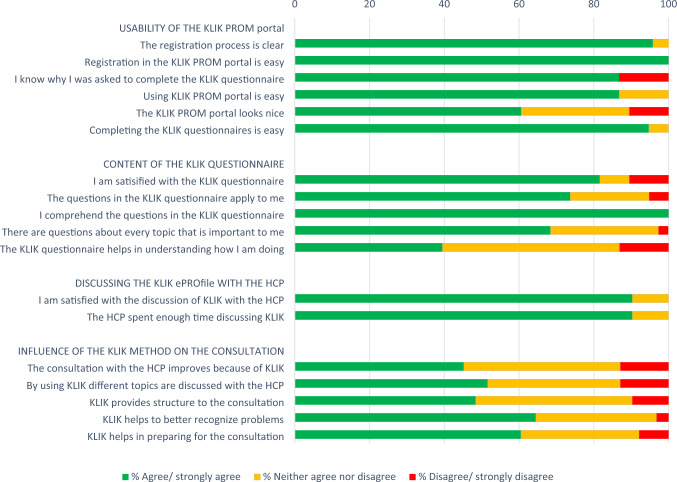
Evaluation of the KLIK PROM portal by childhood cancer survivors who completed the KLIK questionnaire (*N* = 38)

As shown in Fig. 3, the vast majority of CCSs evaluated the usability of the KLIK PROM portal positively (agree/strongly agree), ranging from 86.8 to 100% on the individual items, except for the appearance of the KLIK PROM portal (60.6%). CCSs who disagreed with the statement that the portal looks nice (10.5%), called the portal ‘outdated’ or ‘childish.’ In the interviews it was mentioned that this was because of the bright colors that were used.

#### Content of the KLIK questionnaire

CCSs spent an average of 10 min completing the KLIK questionnaire (range 3–20 min), and most were satisfied with this (94.7%). CCSs were willing to spend a maximum of on average 17 min, and 36.8% reported a maximum of 10 min. Most CCSs (81.6%) reported satisfaction with the KLIK questionnaire. This was the case for the applicability (73.3%), comprehensibility (100%), and completeness (68.4%). No suggestions for additional themes were given. In the interviews, CCSs stated that the attention for their social–emotional well-being was appreciated. CCSs who were less satisfied with the KLIK questionnaire preferred more depth within current topics, e.g., by being able to explain why they had given certain answers. Other CCSs mentioned that the KLIK questionnaire may give a distorted picture as it addresses only the past week instead of a broader time period. In 39.5% of CCSs the questionnaire helped to better understand how they are doing, while for others it did not serve this purpose. Nevertheless, completing the questionnaire could yield additional insight: ‘I think I am always aware of how I am doing, but the questionnaire did give the insight that I am not yet where I want to be.’

#### Discussing the KLIK ePROfile

Most CCSs (81.6%) reported that the KLIK ePROfile was discussed with the HCP during the consultation. Almost all CCSs (> 90%) were satisfied with the conversation and with the amount of time spent discussing the ePROfile.

#### Influence of the KLIK method on the (preparation of the) consultation

Most CCSs (60.5%) reported that the KLIK questionnaire is helpful in preparing for the consultation. In the interviews CCSs mentioned that they systematically reflected on the themes covered in the questionnaire, and that it was helpful to think in advance about questions to ask the HCP.

Almost half of the CCSs (45.2%) felt that the KLIK method improved the conversation and the majority (64.5%) indicated that it helped to better recognize problems*:* ‘You start to realize how you are doing by completing the questionnaire, and so the doctor can help you better with your struggles (..).’ Some reported that little had changed compared to consultations without the KLIK method, while more than half of the CCSs (51.6%) reported that other topics were discussed, such as ‘feelings.’ The KLIK method gave structure to the conversation for almost half of the CCSs (48.4%). A substantial part (41.9%) rated this statement as neutral: ‘Structure has always been there, but it ensures that certain matters are discussed that might otherwise not be discussed.’

In Table 3 the results are summarized in terms of barriers and facilitators.

**Table 3:** Summary Facilitators and barriers of the KLIK method according to childhood cancer survivors (CCSs) and health care practitioners (HCPs)

Themes	Facilitators		Barriers	
	CCSs	HCPs	CCSs	HCPs
*Usability of the KLIK PROM portal*	-Satisfied with the appearance of the KLIK PROM portal	– Satisfied with the appearance of the KLIK PROM portal	Some thought the KLIK PROM portal looked ‘outdated’ or ‘childish’ e.g., because of the bright colors	*Not mentioned*
	– ‘Easy to use’	– ‘Easy to use,’ ‘intuitive’		
		– Quick overview of CCSs’ functioning with traffic light colors and graphs		
*Completing questionnaires in The KLIK PROM portal*	Completing questionnaires in the KLIK PROM portal was easy for most	*Not applicable*	Having to create an account before the consultation appeared a barrier for some	CCSs often do not complete the KLIK questionnaire unless they are reminded to do so
*Time*	Satisfied with the completion time of the KLIK questionnaire	– The KLIK method did not lengthen the consultation	Not all CCSs had time to register and complete questionnaires in the KLIK PROM portal	One HCP needed some extra time to prepare the consultation
		– The quick overview that the KLIK ePROfile provides helps to efficiently address CCSs’ problems		
*Content of the KLIK questionnaire*	– All important topics are covered in the KLIK questionnaire	The KLIK questionnaire provided a quick impression of how CCSs are doing on important life domains	– Some would like to be able to explain, e.g., in an open field why they gave certain answers in the KLIK questionnaire	– Most were in favor of adding a fatigue questionnaire
	– Attention for social–emotional well-being was appreciated		– Time frame of PROM: some preferred the KLIK questionnaire to address a broader time period than just last week	-Most preferred the KLIK questionnaire to address a broader time period than just last week
	– The KLIK questionnaire helped some to gain more insight into their mental health			– Some indicated the need for customization of the questionnaire for CCSs with cognitive disabilities
*Discussing the KLIK ePROfile*	– The KLIK questionnaire was discussed with the HCP during the consultation with most CCS	Helpful to show CCSs the results of the KLIK questionnaire with traffic light colors or graphs	Some did feel the need to discuss the KLIK questionnaire while the HCP did not address it	– CCSs’ questionnaires were not always visible to HCPs as the KLIK team had to complete manual steps after CCSs’ first registration
	– Satisfied with the conversation about KLIK with the HCP			-One HCP would like to receive more training on the discussion of the KLIK questionnaire with CCSs
*Conversation content*	– Other topics were discussed with the use of the KLIK method, such as ‘feelings’	– KLIK facilitates talking in depth on topics of concern	Some felt they could discuss their feelings with their HCP also without the use of the KLIK method, and felt that little had changed	According to HCPs, similar topics are discussed as without using the KLIK method
	– Thinking prior to the consultation about emotional well-being and questions to ask the HCP ensures all important topics are covered during the consultation	– KLIK helps to ensure all topics important to CCSs and HCPs are discussed during the consultation		
		– KLIK could be helpful in opening the conversation as HCPs could refer to the questionnaire when addressing CCSs’ concerns or questions		
*Detecting problems*	Most found that the KLIK method helps to better recognize their problems	*Not mentioned*	*Not mentioned*	*Not mentioned*
*Preparation for the consultation*	The KLIK questionnaire is helpful in preparing for the consultation: CCSs evaluate their functioning on all important life domains and think of questions to ask the HCP	– CCSs are better prepared when visiting the HCP as they have thought in advance about their concerns and questions	Some CCSs also know how they are doing without a questionnaire, but they do experience additional insight by completing the KLIK questionnaire	*Not mentioned*
		– HCPs are better prepared as they have an impression of CCSs’ questions and concerns prior to the consultation		
*Integration of the KLIK method within the organisation*	*Not mentioned*	It was helpful that the KLIK PROM portal is integrated into the electronic system of the Máxima	*Not mentioned*	– Structural support in calling CCSs to remind them of completing the KLIK questionnaire is important
				– It would be efficient if a doctors assistant would call CCSs with a list including all details for the consultation including the use of the KLIK PROM portal

### User satisfaction HCPs

#### Overall satisfaction with the KLIK method

HCPs (*N* = 5) reported on average an overall satisfaction with the KLIK method of 7.5 (range 6–9).

#### Usability of the KLIK PROM portal

All HCPs were satisfied with the appearance of the KLIK PROM portal. Most (4) mentioned that they could see in one glance how CCSs are doing because of the traffic light colors and graphs. All HCPs found KLIK ‘easy to use’ or ‘intuitive,’ and thought it was helpful that the KLIK PROM portal is integrated into the electronic system of the Máxima.

#### Content of the KLIK questionnaire

All HCPs stated that the KLIK questionnaire covers important life domains, although most (3) would add a fatigue questionnaire as fatigue is a common problem among CCSs. Furthermore, most (3) HCPs would like the KLIK questionnaire to address a broader time period than the last week. Some (2) HCPs asked for attention for CCSs with cognitive disabilities, e.g., by providing proxy questionnaires for their parents.

#### Discussing the KLIK ePROfile with CCSs

Most (4) HCPs indicated that they (almost) always discussed the KLIK ePROfile with CCSs. Reasons for not discussing the KLIK ePROfile were: forgotten (2), no problems reported (1), lack of time (1), or many problems had already been discussed without using the KLIK ePROfile (1).

#### Influence of the KLIK method on the (preparation of the) consultation

Most (4) HCPs mentioned that CCSs were better prepared for the consultation if they had completed the KLIK questionnaire because CCSs had then thought in advance about their (mental) health and questions for the HCP. Some (2) HCPs stated that the overview of CCSs’ functioning in the KLIK ePROfile and the possibility to see CCSs’ questions prior to the consultation also helped them as clinicians to better prepare for the consultation.

All HCPs mentioned advantages of the KLIK method in the communication with CCSs. Some (2) found it helpful to show CCSs the results of the KLIK questionnaire with traffic light colors or graphs. For some (2), the KLIK ePROfile was helpful in opening the conversation with CCSs as HCPs could refer to it when addressing CCSs’ concerns and questions. It was also mentioned that the KLIK method provides the opportunity to go in depth on certain topics, e.g., when scores below the norm were noticed. Finally, one HCP mentioned that KLIK can serve as a ‘checklist’ to ensure all topics important to HCPs and CCSs are discussed.

Most (4) HCPs indicated that the use of the KLIK method did not lengthen the consultation as HRQOL topics should be addressed anyway. The quick overview that the KLIK ePROfile provides was mentioned by these HCPs as facilitating to efficiently address CCSs’ problems. For one HCP the preparation took a few minutes longer, but this was acceptable to her.

#### Feeling competent to discuss the KLIK ePROfile

All HCPs mentioned that the KLIK training prepared them sufficiently to work with the KLIK method, although one HCP believed that an additional training on what actions to take when CCSs rate their HRQOL below the norm could be helpful. The other (4) HCPs indicated that they did not need additional training as they had already experience in discussing these topics before using the KLIK method.

#### Support in working with the KLIK method

HCPs were satisfied with the support of the KLIK team and the quick solutions they offered to technical problems. However, some (2) still experienced technical barriers, mainly due to manual steps to take by the KLIK team before first-time-user CCSs could complete the KLIK questionnaire and HCPs could see their answers.

All HCPs stressed the importance of the KLIK team in reminding CCSs to complete the KLIK questionnaire. One HCP mentioned that it would be efficient if a doctors assistant would call CCSs about all issues relevant for the consultation, including the KLIK PROM portal.

In Table 3 the results are summarized in terms of barriers and facilitators.

## Discussion

This study provides insight into the use of PROMs in survivorship care for young adult CCSs, including implementation fidelity, and CCSs’ and HCPs’ satisfaction. In line with the use of the KLIK method in pediatric oncology during active treatment as studied in 2012–2014 [23], registration and completion rates were high (79% and 90% respectively). The percentage of HCPs discussing the KLIK questionnaires during the consultation (75%) was higher than previously found during active treatment (56–62%). The engagement of HCPs in the KLIK implementation team in an early stage was supportive to the implementation, and may have led HCPs to propagate the KLIK method within the organization. Our experiences highlight the importance of involving clinicians in the implementation process of PROMs in clinical practice as well as relying on champions who were enthusiastic and thereby promote the intervention [23, 29]. There is, however, still room for improvement as in our study in only 53% of CCSs the full cycle of registration, completion, and discussion of the KLIK questionnaire was completed.

In line with the literature [24, 25], CCSs and HCPs were satisfied with the KLIK method. KLIK served several benefits, such as improved preparation of the consultation, improved communication between CCSs and HCPs, and improved insight into CCSs’ functioning. CCSs appreciated the attention for their social–emotional well-being. In the literature similar benefits have been reported for the use of PROMs in general in clinical practice in CCSs and other patient groups [12–19]. The results of our study show that the benefits of using PROMs in clinical practice [18, 24–26] also apply to survivorship care for young adults.

With the different methods used (registrations/surveys/interviews) and the different perspectives (CCSs/HCPs) examined, this study yield valuable information for the use of PROMs in clinical practice. Our study reveals several issues important to take in mind when implementing the KLIK method and PROMs in general. First, a supporting team that reminds CCSs of completing the KLIK questionnaire appeared essential. As mentioned in previous research [25], improvement of the completion rate of PROMs is a point of attention and reminders are necessary to engage CCSs. There may be a role for the doctors assistant to facilitate the use of the KLIK method, which can be incorporated in preparations (e.g., telephone contact) for consultations. Furthermore, integration of the KLIK PROM portal within electronic medical records is recommended, so that CCSs could receive automatic reminders via this portal or via email or SMS.

Secondly, based on their experience with the KLIK method, HCPs would like an additional questionnaire about fatigue in the KLIK PROM portal. Initially, in consent with HCPs, only the PedsQL was selected at the start of the implementation, containing one item about fatigue. However, when adding questionnaires, it is important to realize that a substantial part of CCSs is not willing to spend more than 10 min completing questionnaires. A solution may lie in the use of computerized adaptive testing (CAT), which offers questions based on previously given answers [32]. CAT is incorporated in the Patient-Reported Outcomes Measurement Information System (PROMIS) which consists of several item banks measuring components of physical, mental, and social health [33]. PROMIS item banks can be administered via the KLIK PROM portal, and ensure reliable scores based on few questions, so that the burden of completing questionnaires can be relieved. Furthermore, CCSs were satisfied with the content of the KLIK questionnaire, but some preferred more depth within existing topics, e.g., by adding open fields for providing more details. It is, however, important to realize that KLIK is a communication tool between HCPs and CCSs, not intended as a replacement of HCPs asking additional questions during the consultation [20]. Finally, in line with literature [9], HCPs mentioned the importance to customize the KLIK questionnaire to specific CCS groups, in particular those with cognitive disabilities who are unable to complete questionnaires. For this group alternatives should be available such as proxy questionnaires completed by caregivers.

Lastly, the KLIK method could be further tailored to the LATER outpatient clinic for (young) adults. CCSs mentioned that the lay-out of the KLIK PROM portal could be more age-appropriate. Although the option to adjust the appearance of the KLIK PROM portal to one’s personal preference had already been available, an age-appropriate lay-out could be offered by default to adult CCSs. Also, some CCSs and HCPs preferred the KLIK questionnaire to cover a broader time frame instead of just last week, given that most CCSs visit the LATER outpatient clinic once every 1–5 years. However, the time frame should not be restrictive in the discussion of CCS’s functioning because the ePROfile is meant as starting point for the discussion of CCS’s functioning.

Some limitations should be considered. First, the CCSs’ response rate for the evaluation survey was low (18%) so representativeness was not guaranteed. However, still diverse CCSs (regarding use of and satisfaction with the KLIK method) were represented in the survey and in the interviews. A second limitation lies in the rather long time (a few weeks) between the use of the KLIK method and CCSs’ study participation, especially in the interviews. Nevertheless, CCSs reported to remember completing and, if applicable, discussing the KLIK questionnaire. Third, to determine if the KLIK questionnaire was discussed during consultation, we relied on notes of the HCPs. While HCPs were reminded in the training to document the discussion of the KLIK questionnaire, it is possible that some have overlooked this step. However, we asked CCSs in the evaluation surveys if the KLIK questionnaire was discussed which yielded a comparable discussion percentage. Finally, this study focused on the implementation and evaluation of the KLIK method in CCSs aged 18–30 years. Caution should be taken when generalizing the results to CCSs aged ≥ 30 years as in different age groups different life domains and needs can be prominent. Therefore, the next step is to implement and evaluate the KLIK method in CCSs aged ≥ 30 years, in which first the choice of which PROM for which outcomes to be used is key.

To conclude, the KLIK method is a valuable and feasible tool to systematically monitor and discuss HRQOL in survivorship care for young adult CCSs. The benefits of the KLIK method and the use of PROMs in general previously found in pediatric patients and parents, such as increased insight in patient’s functioning and improved preparation before and communication during the consultation, extend to young adult CCSs in survivorship care. Structural support by a PROM team is essential, as well as the assessment of fatigue in CCSs in addition to HRQOL. When selecting questionnaires, it is important to take the perspective of CCSs in mind and the limited time most are willing to spend completing PROMs.

### Supplementary Information

Below is the link to the electronic supplementary material.

Supplementary file1 (DOCX 32 kb)

Supplementary file2 (DOCX 22 kb)

## Data Availability

The data that support the findings of this study are available from the corresponding author upon reasonable request.
