# Supplementary material for: Monitoring health related quality of life in survivorship care of young adult survivors of childhood cancer using web-based patient-reported outcome measures: survivors’ and health care practitioners’ perspectives on the KLIK method
Source: Qual Life Res. 2023 Aug 24;33(1):145–56. doi: 10.1007/s11136-023-03504-z (PMC10784327; doi:10.1007/s11136-023-03504-z)
Supplement: Supplementary file 1 — Supplementary file1 (DOCX 32 kb) [file 11136_2023_3504_MOESM1_ESM.docx]

Appendix A

| **Topics** | **Questions** |
| --- | --- |
| *Discussing the KLIK questionnaire with CCSs* | - I would like to ask you a few questions about the use of KLIK during consultations at the LATER outpatient clinic. Can you tell me about your experience in discussing the KLIK questionnaire with CCSs? - Can you estimate how often you were able to discuss the KLIK questionnaire with CCSs? *Always, usually, occasionally, almost never, never.* If you were not always able to discuss the questionnaire, then what is the reason for that? - What problems do you encounter when using the KLIK PROM portal? What could help solving these problems? - What percentage of the consultation do you approximately spend on discussing the KLIK questionnaire? What do you think about that? - In your experience, what role does the KLIK PROM portal play in identifying psychosocial problems in CCSs? - What influence does the KLIK PROM portal have on the content of the consultation? If any, which other topics are discussed with the use of the KLIK PROM portal? - What do you think are the most important advantages of the KLIK PROM portal? - What do you think are the most important disadvantages of the KLIK PROM portal? |
| *The KLIK PROM portal* | - What do you think of the KLIK PROM portal with regard to a) usability and b) appearance/ layout? - Which parts of the KLIK ePROfile do you mainly feed back to CCSs (literal answers, traffic light colours, graphs)? |
| *The KLIK questionnaire* | - The PedsQL is used to assess HRQOL in CCSs in the KLIK PROM portal. What do you think about the topics discussed in the PedsQL? - Would you like to add other questionnaires to the KLIK PROM portal in addition to the PedsQL? If so, which ones? |
| *Support in working with the KLIK method* | - Before you started working with the KLIK PROM portal you were trained by someone from the KLIK team. What did you think about the training? Did the training provide sufficient preparation in working with the KLIK PROM portal? What were areas for improvement? - How do you experience the support of the KLIK team in working with the KLIK PROM portal, e.g. when you have questions? |
| *General questions* | - Taking everything together, how satisfied are you with the use of the KLIK PROM portal on a scale from 0-10? Explain your answer. What could be done to increase your satisfaction? - Do you have any other comments about the KLIK PROM portal not yet discussed? - Characteristics: previous use of the KLIK PROM portal, age, profession, number of years of experience in survivorship care. |
